# Kompetitive Allele Specific PCR (KASP™) genotyping of 48 polymorphisms at different caprine loci in French Alpine and Saanen goat breeds and their association with milk composition

**DOI:** 10.7717/peerj.4416

**Published:** 2018-02-21

**Authors:** Szilvia Kusza, Ludovic Toma Cziszter, Daniela Elena Ilie, Maria Sauer, Ioan Padeanu, Dinu Gavojdian

**Affiliations:** 1 Research and Development Station for Sheep and Goats Caransebes, Academy for Agricultural and Forestry Sciences, Caransebes, Romania; 2 Institute of Animal Science, Biotechnology and Nature Conservation, University of Debrecen, Debrecen, Hungary; 3 Banat’s University of Agricultural Sciences and Veterinary Medicine ‘King Michael I’ from Timisoara, Timisoara, Romania; 4 Research and Development Station for Bovine Arad, Academy for Agricultural and Forestry Sciences, Arad, Romania

**Keywords:** KASP, Saanen, Polymorphism, Romania, French alpine, Milk traits, Associations

## Abstract

Using a novel and fast genotyping method called Kompetitive Allele Specific PCR (KASP™), we carried out a pilot study on 48 single nucleotide polymorphisms (SNPs) belonging to 40 genes in French Alpine (*n* = 24) and Saanen (*n* = 25) goats reared in Romania. Furthermore, the associations of the 13 polymorphic genetic variants with milk production and composition were investigated. Thirty-five SNPs did not show polymorphism in the studied populations. Polymorphic SNPs were detected in the following genes: *CAST, CLEC4E, DES, GHRHR, HSP90AA1, IL15RA, IL1RN, IL8, MITF, PPRC1, SOCS3, TNF* and *TNFSF13*. The studied Alpine population was in Hardy-Weinberg disequilibrium at the g.62894878A>G locus (rs671391101) (*P* < 0.05). The results showed that four SNPs rs671391101 (*GHRHR*), rs640582069 (*IL1RN*) rs635583012 (*SOCS3*) and rs635969404 (*IL15RA*) out of the 13 polymorphic markers were significantly associated with milk production, protein, fat and lactose content in the Alpine breed. However, no significant effect was recorded in the Saanen population regarding milk yield or milk chemical composition. The current results provide new insights for the development of SNP marker-assisted selection technology in the goat industry and confirm the potential of using SNPs for the* GHRHR*, *IL1RN*, *SOCS3*, and *IL15RA* genes as candidate genes for selection, highlighting the direct implications of such genes for farm production outputs. The results from this study are relevant for future goat genomic studies and the inclusion of the associated traits into up-to-date selection schemes.

## Introduction

The goat sector within the European Union accounts for 12.3 million head (EUROSTAT, 2016), concentrated geographically in Greece, Spain, Romania, France and Italy (10.68 million altogether), with the farmers essentially focused on dairy production (milk and cheese production). According to Eurostat reports for the year 2016, Romania alone holds a flock of 1.48 million breeding goats, representing approximately 12% of the goats reared in the EU. As in other European countries, the main focus of the sector is milk and cheese production, with goat meat being considered a marginal product. Milk production traits are of fundamental importance in livestock production and are strongly related to the efficiency and economy of the farm (Erhardt et al., 2010; An et al., 2011). However, there are significant differences in the average milk production of goats between regions, mostly due to management and feeding systems and to genetic background (breed, selection). In Romania, goats are reared predominantly in extensive low-input production systems (Raducuta, Calin & Purdoiu, 2012), and the unimproved indigenous Carpatina breed represents over 90% of the population. The breed has modest production outputs, with milk yields estimated at 220 to 350 kg/lactation (Padeanu, 2001, Pascal et al., 2011).

Consequently, farmers started to import Saanen and French Alpine goats, the main specialized dairy goat breeds reared worldwide. The milk yield estimated for the two breeds ranges between 800 to 1100 kg/lactation when they are reared in intensive production systems (Palhiere et al., 2014). Both the Saanen and Alpine breeds are reared as purebreds where large commercial farms are concerned, while on small- and medium-sized farms, which practice more extensive rearing, the bucks are used for crossbreeding with the indigenous Carpatina breed in order to improve their milk yield and udder conformation.

The ongoing growth and increasing accessibility of genetic and genome sequencing data has given new perspectives for research in domestic ruminant species (Dong et al., 2013; Elsik et al., 2009). Polymorphism of milk proteins, which has been linked to the chemical composition and biological characteristics of milk, as well as the processing properties of cheese, is well studied and documented worldwide (Martin et al., 2002; Vinesh et al., 2013). Selection can be carried out to increase the frequency of alleles with a positive effect on a given production trait (Dekkers, 2004). Variations in these genes, which show strong associations with economically important production traits, such as traits related to milk production, are useful tools for marker-assisted selection (Parmentier et al., 2011). In the case of dairy goats, the effect of caseins—especially that of α_s1_ casein (*CSN1S1*)—on milk composition (mainly protein content) has received significant attention from both geneticists and animal scientists in the last decade. Moreover, the state of knowledge on goat genetics has been significantly improved from the year 2010 onward, subsequent to the sequencing of the goat genome and the release of the goat SNP50, which allowed several genome-wide association studies (GWAS) (Martin et al., 2016). The aim of the current pilot research was to investigate the polymorphism of 48 SNPs of 40 genes on dairy production and major milk components using genotyping methods in French Alpine and Saanen goats reared in Romania. Furthermore, association study was performed between milk traits and polymorphic SNPs. The ultimate purpose was to find and describe polymorphisms that could prove useful for the design of future genetic improvement schemes in the two dairy breeds.

## Materials & Methods

### Animals and management

Forty-nine genetically unrelated multiparous goats (3rd lactations) reared under intensive commercial conditions (Northern Romania: 47°34′15″N 23° 25′41″E), including twenty-five French Saanen and 24 French Alpine individuals, were included in this study. The commercial farm manages a total of 1035 breeding goats in a mixed-breed system, with both Saanen and Alpine being reared under identical management and feeding conditions.

At the farm level, both the Saanen and Alpine herds have been selected predominantly to increase milk yield, with the main selection aim being set at a production of 5,000 kg of milk per productive life. The animals selected for the current study were in the top 20% for milk yield, and were designated to produce the replacement does and bucks. All does were inseminated with frozen semen imported from France and Austria in order to avoid inbreeding within the populations. The does taken into the study were descendants of purebred animals introduced into Romania in 2009, following an import of Saanen and Alpine goats from France, representing a diverse sampling of genetic lines (12 lines/breed unit). Reproductive failure and udder-related problems were the criteria normally used to define which does would be culled. The culling rate at the farm level was 25%, with a conception rate of 83% and an average prolificacy of 186%. Pregnancy scanning was practised starting on day 45 after artificial insemination.

All animals were included in the Official Performance and Recording Scheme, being registered as nucleus reference breeding herds, which produce and disseminate tested bucks. Does were milked twice per day in a ‘herringbone’ milking parlour (2 sides × 24 units). The average lactation length on the studied farm was 280 ± 15 days. The following production data were recorded for the studied animals: milk yield (kg), fat (%), protein (%) and lactose (%).

Does were kept on a straw bedding, with a space allowance of 1.7 m^2^ per head in the resting area and free access to water and outdoor paddocks of 4.5 m^2^/goat. They received a daily feed ration made of high-quality alfalfa hay *ad libitum* and pelleted feed (16% CP). Does were housed in groups of 40 to 50 animals, according to lactation stage and productivity, regardless of their breed.

Hair follicles were taken from each animal and kept at 4 °C until further processing. The research activities were performed in accordance with the European Union’s Directive for animal experimentation (Directive 2010/63/EU).

### Selection of SNPs

A total of 48 single nucleotide polymorphisms (SNPs) of 40 genes have been chosen based on the results of prior genome-wide association study (GWAS) and marker assisted selection study across the goat genome (An et al., 2011; Boulanger, Grosclaude & Mahé, 1984; Martin et al., 2002; Martin et al., 2016; Mousavizadeh et al., 2009; Singh et al., 2015; Tabaran et al., 2014; Vlaic et al., 2010; Zaulet et al., 2008) (Table 1). Caprine SNP data were collected from the Single Nucleotide Polymorphism Database, or dbSNP (NCBI). Special focus was given to the major genes associated with milk production and composition traits, found at different chromosome localizations.

### Genomic DNA extraction and genotyping

Genomic DNA was isolated using a method described by the FAO/IAEA (2004). The DNA was stored at −20 °C until further analysis. DNA concentration was measured with a NanoDrop Spectrophotometer (Thermo Scientific, Waltham, MA, USA). All samples were diluted to a uniform concentration, and the equivalent of 50 ng of DNA per sample was used for genotyping. Kompetitive Allele Specific PCR (KASP™, LGC Genomics, Teddington, Middlesex, UK) genotyping was used for the bi-allelic discrimination of the selected 48 SNPs (Table 1). Results were visualized with SNP Viewer software (version 1.99, Hoddesdon, UK). Genotype data for each animal were exported for the statistical analysis.

### Data analysis

The raw allele calls received from LGC Genomics were analysed with KlusterCaller software from LGC Genomics. Further, a genetic association study was conducted on polymorphic SNPs.

To determine genotype frequencies for all polymorphic SNPs and estimate the effect of SNP polymorphism and breed on milk yield and composition, we performed factorial ANOVA using the statistical software Statistica (StatSoft, Tulsa, OK, USA) with the following model:

\begin{eqnarray*}{y}_{ijk}=\mu +SN{P}_{i}+BREE{D}_{j}+SNPxBREE{D}_{ij}+{\epsilon }_{ijk} \end{eqnarray*}

where: *y*_*ijk*_ is the milk yield, fat percentage, protein percentage or lactose percentage; µ is the overall mean; *SNP*_*i*_ is the effect of the selected SNP polymorphism (with 1, 2 or 3 levels, depending on the studied SNP); *BREED*_*j*_ is the breed effect (Saanen or Alpine); *SNPxBREED*_*ij*_ is the interaction between SNP polymorphism and breed; and *ϵ*_*ijk*_ is the random error.

The *chi-squared* test (*χ*^2^) was used to determine whether the populations were in Hardy-Weinberg equilibrium (HWE). HWE analysis was performed for setting the genetic association studies.

The significance of each main effect (SNP and breed) as well as that of the interaction between main factors was verified using Shapiro–Wilk’s test (Hill & Lewicki, 2007). The significance of differences between least-squares means was tested using Tukey contrasts.

For each SNP, only the animals found to have been genotyped for that SNP were taken into consideration. This is why the number of individuals varied across the SNPs.

**Table 1:** Selected SNPs used in the study for genotyping the Saanen and French Alpine goat breeds.

SNP	Locus	Gene name	Allele substitution	Position	Chromosome
rs646939931^a^	BLG	beta lactoglobulin	C/G	101688547	11
rs666806435	CAST	calpastatin	C/T	92154468	7
rs669986850	CLEC4E	C-type lectin domain family 4, member E	C/T	93538087	5
rs640597911^a^	CLEC4E	C-type lectin domain family 4, member E	A/G	93531862	5
rs647010244^a^	CSN1S2	alphas2 casein	C/T	82783242	6
rs655570397^a^	CSN2	beta casein	C/G	82706566	6
rs268293109^a^	CSN3	K-casein	A/G	82905979	6
rs671256096	DES	desmin	C/T	106743716	2
rs666579277^a^	DGAT1	diacylglycerol O-acyltransferase 1	C/T	11262510	14
rs637389720^a^	DGAT1	diacylglycerol O-acyltransferase 2	A/G	11265136	14
rs662251196^a^	GHR	growth hormone receptor	C/T	31638980	20
rs671391101	GHRHR	growth hormone releasing hormone receptor	A/G	62894878	4
rs660778229	HSP90AA1	heat shock protein 90 kDa alpha (cytosolic), class A member 1	A/G	63869868	21
rs648330532^a^	HSP90AA1	heat shock protein 90 kDa alpha (cytosolic), class A member 1	C/G	63869848	21
rs657411574^a^	HSPA12B	heat shock 70 kD protein 12B	A/G	49737502	13
rs642665135^a^	HSPA13	heat shock protein 70 kDa family, member 13	A/G	21576446	1
rs644393790^a^	ICOSLG	inducible T-cell co-stimulator ligand	C/T	141880385	1
rs655800693^a^	IGF1	insulin-like growth factor 1	G/T	64133586	5
rs635969404	IL15RA	interleukin 15 receptor, alpha	C/T	10354813	13
rs640194180^a^	IL1B	interleukin 1, beta	A/G	46047709	11
rs640582069	IL1RN	interleukin 1 receptor	G/T	46353777	11
rs650731617^a^	IL4R	interleukin-4 receptor	C/T	24974944	25
rs646307174^a^	IL6	interleukin-6	C/T	29257937	4
rs665173888	IL8	interleukin 8	A/G	86040123	6
rs655587739^a^	LIPE	lipase E, hormone sensitive type	A/G	49565649	18
rs657028280^a^	LYZL6	lysozyme like 6	A/G	44356928	19
rs651811571^a^	MAP3K14	mitogen-activated protein kinase kinase kinase 14	C/G	44062724	19
rs668924310^a^	MAP3K14	mitogen-activated protein kinase kinase kinase 14	C/T	44050352	19
rs657011040^a^	MC5R	melanocortin 5 receptor	C/G	43663391	24
rs651461190^a^	MC5R	melanocortin 5 receptor	C/T	43665490	24
rs654112183^a^	MITF	melanogenesis associated transcription factor	C/T	31431210	22
rs664283580	MITF	melanogenesis associated transcription factor	C/T	31429293	22
rs641851617^a^	MSTN	myostatin	A/G	6314057	2
rs671509714	PPRC1	peroxisome proliferator-activated receptor gamma, coactivator-related 1	A/G	21172380	26
rs668650719^a^	PRLR	prolactin receptor	G/T	38802036	20
rs665266215^a^	PRNP	prion protein	G/T	45240766	13
rs268292980^a^	PRNP	prion protein	A/G	45240888	13
rs641385611^a^	PTX3	ventricular zone expressed PH domain-containing 1	A/G	108081185	1
rs662591991^a^	SLC10A5	solute carrier family 10, member 5	A/T	90969357	14
rs643200957^a^	SLC11A1	solute carrier family 11 (proton-coupled divalent metal ion transporters), member 1	C/T	105763369	2
rs666944028^a^	SLC11A1	solute carrier family 11 (proton-coupled divalent metal ion transporters), member 1	C/T	105766113	2
rs635583012	SOCS3	suppressor of cytokine signaling 3	G/T	52626440	19
rs639895207^a^	TGFB1	transforming growth factor, beta 1	C/T	49347870	18
rs661914424^a^	TLR3	toll-like receptor 3	C/G	14987931	27
rs669391945^a^	TLR4	toll-like receptor 4	A/C	105007687	8
rs661165283	TNF	tumor necrosis factor	A/T	26141981	23
rs669561078	TNFSF13	tumor necrosis factor (ligand) superfamily, member 13	A/G	26523480	19
rs638567475^a^	ZP2	zona pellucida glycoprotein 2	A/G	19021217	25

**Notes.**

a non-polymorphic loci.

## Results

For all investigated SNPs the KASP assays produced 2,230 identified allele calls and 122 unidentified allele calls with an allele call rate of 94,81% and a mean of unidentified allele calls of 5.19%. A total of 13 SNPs (27.08%) among the selected 48 SNPs were polymorphic across the two populations and further used for the association study (Table 2). The polymorphic SNPs were found in the following genes: *CAST, CLEC4E, DES, GHRHR, HSP90AA1, IL15RA, IL1RN, IL8, MITF, PPRC1, SOCS3, TNF* and *TNFSF13*. Although the set of SNPs was selected from dbSNP, a large number of SNPs (*n* = 35, 72.92%) were not polymorphic and were therefore excluded from the association study. Thirty-five SNPs were not polymorphic (Table 1).

**Table 2:** List of polymorphic SNPs and genotypes frequencies in Saanen and French Alpine goat breeds.

			Genotype frequencies		
SNP	Allele substitution	Genotype	Saanen	French Alpine	Total population
rs666806435	C>T	CC	0.72	0.91	0.81
		CT	0.24	0.09	0.17
		TT	0.04	0	0.02
N			25	22	47
rs669986850	C>T	TT	0.83	0.53	0.70
		TC	0.17	0.47	0.30
		CC	0	0	0
N			24	19	43
rs671256096	C>T	CC	0.83	0.96	0.89
		CT	0.17	0.04	0.11
		TT	0	0	0
N			24	22	46
rs671391101	A>G	AA	1.00	0.89	0.95
		GG	0	0.11	0.05
		AG	0	0	0
N			25	18	43
rs660778229	A>G	AA	0	0.10	0.04
		GG	0.76	0.66	0.72
		AG	0.24	0.24	0.24
N			25	21	46
rs635969404	C>T	CC	0.24	0.59	0.40
		TC	0.44	0.32	0.38
		TT	0.32	0.09	0.22
N			25	22	47
rs640582069	G>T	TG	0.40	0.50	0.45
		GG	0.52	0.37	0.45
		TT	0.08	0.13	0.10
N			25	24	49
rs665173888	A>G	AA	0	0.05	0.02
		GG	0.80	0.69	0.75
		AG	0.20	0.26	0.23
N			25	19	44
rs664283580	C>T	CC	0.92	0.96	0.94
		TC	0.08	0.04	0.06
		TT	0	0	0
N			25	24	49
rs671509714	A>G	GG	1.00	0.91	0.96
		AG	0	0.09	0.04
		AA	0	0	0
N			25	21	46
rs635583012	G>T	TG	0.68	0.50	0.60
		GG	0.16	0.18	0.17
		TT	0.16	0.32	0.23
N			25	22	47
rs661165283	A>T	AA	0.12	0	0.06
		AT	0.40	0.25	0.33
		TT	0.48	0.75	0.61
N			25	24	49
rs669561078	A>G	AA	0.84	0.85	0.84
		AG	0.16	0.15	0.16
		GG	0	0	0
N			25	20	45

**Notes.**

N number of studied animals

Among the polymorphic SNPs, two were located in introns (rs660778229-HSP90AA1 and rs635583012-SOCS3), two in the 3′ UTRs (rs671256096-DES and rs664283580-MITF), and one was an upstream variant (rs669986850-CLEC4E). In addition, eight SNPs were missense substitutions (rs666806435-CAST, rs671391101-GHRHR, rs635969404-IL15RA, rs640582069-IL1RN, rs665173888-IL8, rs671509714-PPRC1, rs661165283-TNF and rs669561078-TNFSF13).

The different genotype frequencies are shown in Table 2. The results of our study outlined that homozygous genotypes were more frequent than heterozygous in most cases. Only the Alpine goats were in Hardy-Weinberg disequilibrium at the g.62894878A>G locus (rs671391101) (*P* < 0.05).

The studied individuals’ genotypes were analysed for association with milk yield and composition in the Saanen and Alpine breeds and in both populations together. The four SNPs—rs671391101 (*GHRHR*), rs640582069 (*IL1RN*) rs635583012 (*SOCS3*) and rs635969404 (*IL15RA*)—had significant effects on milk production, protein, fat and lactose in the Alpine breed and in both populations collectively (Table 3). No significant effects on investigated traits were found for any other polymorphic SNP (results not presented). No significant effects were recorded in the Saanen population regarding milk production, protein, fat or lactose content. Milk yield and fat percentage did not show any significant association with genotype in either the Saanen or the Alpine breed. In the case of total population, the g.46353777T>G SNP located in the *IL1RN* gene was associated with milk production; the g.62894878A>G SNP in the *GHRHR* gene, the g.10354813C>T SNP in the *IL15RA* gene and the g.46353777T>G SNP in the *IL1RN* gene were associated with fat percentage (*P* < 0.05); the g.46353777T>G SNP in the *IL1RN* gene with protein percentage (*P* < 0.05); and the g.52626440T>G SNP in the *SOCS3* gene with lactose percentage. Out of these associations, the g.52626440T>G (rs635583012) SNP located in the intron of the *SOCS3* gene and the g.46353777T>G (rs640582069) SNP located in the *IL1RN* gene were associated with lactose percentage; the g.62894878A>G SNP in the *GHRHR* gene and the g.46353777T>G SNP located in the *IL1RN* gene were associated with protein percentage in the Alpine population (*P* < 0.05). The current findings also underline the significant differences found between the Saanen and Alpine goat breeds based on all 13 markers for milk yield (*P* ≤ 0.05).

**Table 3:** List of SNPs found to be associated with milk yield and composition in Saanen and French Alpine goats.

			Saanen population					French Alpine population					Total population				
SNP	Gene	Geno- type	Genotype frequencies	Milk yield (kg)	Milk fat (%)	Milk protein (%)	Milk lactose (%)	Genotype frequencies	Milk yield (kg)	Milk fat (%)	Milk protein (%)	Milk lactose (%)	Genotype frequencies	Milk yield (kg)	Milk fat (%)	Milk protein (%)	Milk lactose (%)
rs671391101	GHRHR	AA	1.00	874.32 ± 36.81^∗^	3.83 ± 0.03	3.62 ± 0.03	4.19 ± 0.01	0.89	1,039.06 ± 46.01^a∗^	3.74 ± 0.04^a^	3.63 ± 0.03^a^	4.17 ± 0.01^a^	0.95	938.61 ± 31.04^a^	3.80 ± 0.03^a^	3.63 ± 0.02^a^	4.18 ± 0.01^a^
		GG	0					0.11	963.5 ± 130.15^a^	4.02 ± 0.13^a^	3.89 ± 0.1^b^	4.12 ± 0.05^a^	0.05	963.5 ± 130.15^a^	4.02 ± 0.13^b^	3.89 ± 0.10^a^	4.12 ± 0.05^a^
		AG	0					0					0				
rs635969404	IL15RA	CC	0.24	859.83 ± 71.34^a^	3.79 ± 0.07^a^	3.60 ± 0.06^a^	4.20 ± 0.07^a^	0.59	975.15 ± 48.47^a^	3.80 ± 0.05^a^	3.66 ± 0.04^a^	4.23 ± 0.04^a^	0.40	938.73 ± 28.83^a^	3.79 ± 0.04^ab^	3.64 ± 0.04^a^	4.22 ± 0.04^a^
		TC	0.44	829.9 ± 52.69^a^*	3.80 ± 0.05^a^	3.59 ± 0.04^a^	4.20 ± 0.05^a^	0.32	1, 096.14 ± 66.05^a∗^	3.67 ± 0.07^a^	3.62 ± 0.05^a^	4.25 ± 0.06^a^	0.38	933.44 ± 61.16^a^	3.75 ± 0.05^a^	3.61 ± 0.04^a^	4.22 ± 0.04^a^
		TT	0.32	946.25 ± 61.78^a^	3.80 ± 0.06^a^	3.67 ± 0.05^a^	4.16 ± 0.06^a^	0.09	1, 150.00 ± 123.57^a^	3.97 ± 0.13^a^	3.80 ± 0.11^a^	4.17 ± 0.12^a^	0.22	987.00 ± 52.40^a^	3.92 ± 0.06^b^	3.70 ± 0.05^a^	4.16 ± 0.02^a^
rs640582069	IL1RN	TG	0.40	907.20 ± 55.26^a^	3.88 ± 0.06^a^	3.61 ± 0.04^a^	4.21 ± 0.04^a^	0.50	1,021.08 ± 50.44^a^	3.73 ± 0.05^a^	3.65 ± 0.04^ab^	4.24 ± 0.04^a^	0.45	969.31 ± 33.96^ab^	3.80 ± 0.05^ab^	3.63 ± 0.04^ab^	4.23 ± 0.03^a^
		GG	0.52	821.92 ± 48.46^a^*	3.78 ± 0.05^a^	3.61 ± 0.04^a^	4.19 ± 0.04^a^	0.37	1,005.33 ± 58.24^a^*	3.72 ± 0.06^a^	3.58 ± 0.05^a^	4.14 ± 0.05^a^	0.45	896.95 ± 46.05^a^	3.76 ± 0.03^a^	3.60 ± 0.02^a^	4.17 ± 0.02^a^
		TT	0.08	1050.5 ± 123.56^a^	3.89 ± 0.13^a^	3.75 ± 0.1^a^	4.10 ± 0.1^a^*	0.13	1,080.33 ± 100.89^a^	3.95 ± 0.11^a^	3.81 ± 0.08^b^	4.46 ± 0.08^b^*	0.10	1068.40 ± 50.53^b^	3.93 ± 0.11^b^	3.79 ± 0.08^b^	4.32 ± 0.15^a^
rs635583012	SOCS3	TG	0.68	875.35 ± 44.23^a^*	3.80 ± 0.04^a^	3.61 ± 0.03^a^	4.17 ± 0.03^a^	0.50	1,060.00 ± 54.99^a^*	3.81 ± 0.06^a^	3.66 ± 0.04^a^	4.19 ± 0.04^a^	0.60	947.89 ± 41.80^a^	3.81 ± 0.04^a^	3.62 ± 0.03^a^	4.18 ± 0.01^a^
		GG	0.16	868.00 ± 91.19^a^	3.89 ± 0.1^a^	3.62 ± 0.08^a^	4.23 ± 0.07^a^	0.18	1068.75 ± 91.19^a^	3.71 ± 0.1^a^	3.64 ± 0.08^a^	4.09 ± 0.07^a^	0.17	968.37 ± 64.10^a^	3.80 ± 0.07^a^	3.65 ± 0.07^a^	4.16 ± 0.04^a^
		TT	0.16	876.25 ± 91.19^a^	3.87 ± 0.1^a^	3.66 ± 0.08^a^	4.24 ± 0.07^a^	0.32	959.28 ± 68.93^a^	3.75 ± 0.07^a^	3.68 ± 0.06^a^	4.39 ± 0.05^b^	0.23	929.09 ± 35.47^a^	3.80 ± 0.04^a^	3.68 ± 0.04^a^	4.34 ± 0.09^b^

**Notes.**

Column means with different letter superscript differ significantly at *P* ≤ 0.05, within the same SNP

Row means for the same trait marked with * differ significantly at *P* ≤ 0.05, between breeds within genotype

## Discussion

The Kompetitive Allele Specific PCR genotyping assay has been successfully used in order to genotype different organisms (He, Holme & Anthony, 2014; Semagn et al., 2014; Landoulsi et al., 2017). In our work KASP technology was used in Saanen and Alpine goats reared in Romania in order to investigate the polymorphism of 48 SNPs of 40 genes that were previously reported to be associated with the production traits or that potentially contributed to important metabolic conditions. The KASP genotyping system used in the present study generated a high assay success rate (94,81%) and offered a highly locus specificity and accurate genotyping solution. Ours results are in accordance with previously reported KASP assay success rate (Semagn et al., 2014). Furthermore, according to previous studies the KASP genotyping system has low-labour and economic advantages over other genotyping assays (Landoulsi et al., 2017).

The results of this study showed that only 13 of the studied SNPs were polymorphic. The genetic variants *CC* of rs669986850, *TT* of rs671256096, *AG* of rs671391101, *TT* of rs664283580, *AA* of rs671509714 and *GG* of rs669561078 were not observed in the Saanen or the Alpine. The missing genotypes may be attributable to the relatively small number of animals studied.

The results of the current study showed that the homozygous genotypes were more frequent than heterozygous in most cases. All results were tested for deviations from HWE that was used as a means of quality control. Only the Alpine breed was in Hardy-Weinberg disequilibrium at the g.62894878A>G locus (rs671391101) (*P* < 0.05) and therefore this locus raises question for further study on large number of samples. The Hardy-Weinberg disequilibrium for this SNP in French Alpine may be attributable to either selection or the genotyping error of KASP assays that produced 18 identified allele calls and 6 unidentified allele calls. Among the polymorphic SNPs, two were located in introns (rs660778229, rs635583012), and those variants have recently been demonstrated to be significantly associated with quantitative traits in different species, despite the fact that they do not change amino acids (Clark et al., 2015; Maitra et al., 2014; Sun et al., 2015; Zhao et al., 2013; Zhou et al., 2016). The results obtained here on single SNP analyses confirmed the previous findings, since it indicated that the goat *SOCS3* g.52626440T>G beside *GHRHR* g.62894878A>G, *IL1RN* g.46353777T>G and *IL15RA* g.10354813C>T might play an important role in affecting milk production traits and could be linked to major genes that affect milk production traits in goats. Current findings revealed the significant association and the effects of those loci and the investigated milk traits. It was surprising since those genes are not milk protein genes or closely related to them.

*SOCS3* is a member of the suppressors of cytokine signalling (*SOCS*) family of proteins that has a negative effect on cytokine signalling (Krebs & Hilton, 2001). However, polymorphism of *SOCS3* gene was associated with somatic cell score trait in cattle (Strillacci et al., 2014; Wang et al., 2015), mammary development pathway (Raven et al., 2014) and is expressed in goat milk fat globules in response to experimental intramammary infection with *Staphylococcus aureus* (Strillacci et al., 2014; Wang et al., 2015).

Growth hormone (*GH*) was found to have wide range effect on a variety of physiological parameters such as lactation, reproduction, growth traits and metabolism (Mousavizadeh et al., 2009). Marques et al. (2006) detected high levels of polymorphisms at the five exons and the 5′-UTR and 3′-UTR regions of growth hormone gene in Serra da Estrela ewes and found that the two copies of the ovine *GH* gene (namely, *GH2-N* and *GH2-Z*) genotypes significantly affect milk yield in the breed. In contrast, significant differences in milk traits included milk yield, fat, protein, casein and lactose percentage were detected among Sarda sheep genotypes at polymorphic loci only for the *GH2-Z* gene (Dettori et al., 2015). However, there are no information about polymorphism and its associations with milk production traits in goats. Polymorphism and association study of exon 2 and exon 3 of the growth hormone gene with growth traits in goats from two Indian breeds did not find any significant association between genotypes and body development traits (Singh et al., 2015).

In the current study, two less-studied interleukins in goats were found to have a significant association with milk related traits. The *IL1RN* had also significant SNP for milk yield and located on chromosome 11 like a whey protein, β-lactoglobulin gene in goats. However, genetic variants of *BLG* gene involved this study (rs646939931) was not polymorph. Members of the *IL-1* family are pleiotropic cytokines that have important rules in inflammation, immunoregulation and other homeostatic functions in the body (Mora & Weigert, 2016). Marcos-Carcavilla et al. (2007) studied the *IL-1* family members as candidate genes for scrapie susceptibility detection in sheep. However, no studies on goats were published up-to-date. *IL-15γ*, *IL-15Rβ* and *IL-15Rα* are composing the chains of the interleukin-15 receptor (*IL-15R*). In human interleukin-15 receptor alpha is a high affinity IL-15 binding protein that is crucial for mediating IL-15 functions such as memory CD8 T, cell proliferation and NK, NK/T cell, and intestinal intraepithelial lymphocyte development (Schluns, Stoklasek & Lefrançois, 2005). Moreover, no previous study was performed for polymorphism or association studies with production traits in farm animals.

## Conclusion

In summary, this study validated a KASP genotyping assays in goat that can replace the different genotyping assays developed for marker-assisted selection. The validated KASP genotyping assay used for the 48 SNPs will be helpful for diverse applications in goat breeding programs. Furthermore, preliminary results of the current study shows evidence of significant associations between four SNPs located on four genes, which were poorly studied before within the genetic variants and milk production traits in Alpine goats, indicating the potential role of *SOCS3, GHRHR, IL1RN* and *IL15RA* variants on relevant traits in dairy production.

##  Supplemental Information

10.7717/peerj.4416/supp-1 Data S1 Raw data

## Additional Information and Declarations

### Data Availability

The following information was supplied regarding data availability: The raw data has been provided as a Supplemental File.
